# Horizontal transfer of transposons between and within crustaceans and insects

**DOI:** 10.1186/1759-8753-5-4

**Published:** 2014-01-29

**Authors:** Mathilde Dupeyron, Sébastien Leclercq, Nicolas Cerveau, Didier Bouchon, Clément Gilbert

**Affiliations:** 1 Université de Poitiers, UMR CNRS 7267 Ecologie et Biologie des Interactions, Equipe Ecologie Evolution Symbiose, 86022 Poitiers, Cedex, France; 2 Present address: Courant Research Center Geobiology, Geomicrobiology and Symbiosis Group, University of Göttingen, Goldschmidtstraße 3, 37077 Göttingen, Germany

**Keywords:** Horizontal transfer, Transposable elements, Isopod crustaceans, Hexapods

## Abstract

**Background:**

Horizontal transfer of transposable elements (HTT) is increasingly appreciated as an important source of genome and species evolution in eukaryotes. However, our understanding of HTT dynamics is still poor in eukaryotes because the diversity of species for which whole genome sequences are available is biased and does not reflect the global eukaryote diversity.

**Results:**

In this study we characterized two *Mariner* transposable elements (TEs) in the genome of several terrestrial crustacean isopods, a group of animals particularly underrepresented in genome databases. The two elements have a patchy distribution in the arthropod tree and they are highly similar (>93% over the entire length of the element) to insect TEs (Diptera and Hymenoptera), some of which were previously described in *Ceratitis rosa* (*Crmar2*) and *Drosophila biarmipes* (*Mariner-5_Dbi*). In addition, phylogenetic analyses and comparisons of TE versus orthologous gene distances at various phylogenetic levels revealed that the taxonomic distribution of the two elements is incompatible with vertical inheritance.

**Conclusions:**

We conclude that the two *Mariner* TEs each underwent at least three HTT events. Both elements were transferred once between isopod crustaceans and insects and at least once between isopod crustacean species. *Crmar2* was also transferred between tephritid and drosophilid flies and *Mariner*-*5* underwent HT between hymenopterans and dipterans. We demonstrate that these various HTTs took place recently (most likely within the last 3 million years), and propose iridoviruses and/or *Wolbachia* endosymbionts as potential vectors of these transfers.

## Background

Horizontal transfer (HT) of genetic material is the transmission of DNA between non-mating organisms [1]. Most known eukaryote-to-eukaryote HT events are transfers of transposable elements (TEs) [2]. Given the profound impact TEs have on the genome architecture of their hosts, HT of TEs (HTT) is increasingly recognized as an important force in eukaryote genome evolution [3]. On the TE side, spreading between genomes via HT may be viewed as a strategy to escape vertical extinction due to purifying selection, mutational decay and/or host defense mechanisms. Among the over 330 cases of eukaryote-to-eukaryote HTT events characterized so far, the vast majority involve DNA transposons (n = 188 cases) and LTR retrotransposons (n = 118 cases) [4], indicating that the long-term survival of these TEs may rely more on HT than that of non-LTR retrotransposons. Yet, while whole genomes are sequenced at an exponential pace, the global diversity of eukaryote genomes is still poorly represented, precluding any strong generalization on HTT dynamics. Even in animals, whole genome sequencing efforts are biased towards species closely related to model organisms or species of economic interest, and whole genome sequences are lacking for many large taxonomic groups. Our current understanding of the global HTT dynamics and impacts is therefore incomplete, both at the host and TE level.

With only one genome fully sequenced [5] out of over 50,000 species described [6], crustaceans are particularly underrepresented in genome databases. The order Isopoda (Vericrustacea clade according to [7]) is unique among crustaceans in that the colonization of landmasses by one of its lineages (belonging to the suborder Oniscidea) during the Mesozoic yielded a large diversity of terrestrial species (>3,600 [8]) now distributed all over the world in every biotope (except for the poles) [9]. In this study we report new cases of HTT involving terrestrial isopod crustaceans and hexapods. We used a combination of cross-species PCR screening of TEs, phylogenetic and other evolutionary analyses to characterize in detail these HTT and to shed light on the evolutionary dynamics of the first two TEs described in isopod crustaceans.

## Results and discussion

### Characterization of two *Mariner* elements in the isopod crustacean *Armadillidium vulgare*

In order to detect TEs that underwent horizontal transfer between isopod crustaceans and other taxa, we used all consensus sequences deposited in Repbase [10] as of May 2013 as queries to perform BLASTn searches on draft genomic contigs and on a transcriptome of the pill bug *Armadillidium vulgare* that have been generated in our lab as part of other ongoing projects. Importantly, the contigs generated by these projects are too short to carry out a comprehensive *de novo* mining of *A. vulgare* TEs. The BLASTn searches yielded two TEs belonging to the Tc1/Mariner superfamily of Class II DNA transposons that show more than 90% identity over more than 500 bp to *A. vulgare* sequences. The first one (*Crmar2*) was originally characterized in the tephritid fly *Ceratitis rosa* based on a PCR/sequencing screening [11], and the second one (*Mariner-5_Dbi*) was described by Kojima and Jurka [12] in *Drosophila biarmipes* based on whole genome sequence data mining. We reconstructed an *A. vulgare* consensus sequence of both elements (named *Crmar2_Avul* and *Mariner-5_Avul*) using 100 to 1300 bp-long fragments resulting from our various BLAST outputs, such that the entire sequence of the consensus was covered by at least five different copies. *Crmar2_Avul* is 1304 bp in length, has 39-bp terminal inverted repeats (TIRs) and encodes a 361 amino acid (aa) transposase while *Mariner-5_Avul* is 1013 bp in length, has 28-bp TIRs and encodes a 200 aa transposase. Both elements are flanked by TA target site duplications, which is characteristic of the Tc1/Mariner superfamily [13]. The evolution of this superfamily has yielded a large number of elements which have colonized the genome of many eukaryote taxa [14,15] and have been classified in various subfamilies (for example, [16]). *Crmar2* belongs to the *rosa* subfamily [11] and our phylogenetic analysis of the transposase revealed that *Mariner-5_Dbi* belongs to the irritans subfamily [see Additional file 1: Figure S1].

### Taxonomic distribution of the two *Mariner* elements in eukaryotes

**Figure 1:**
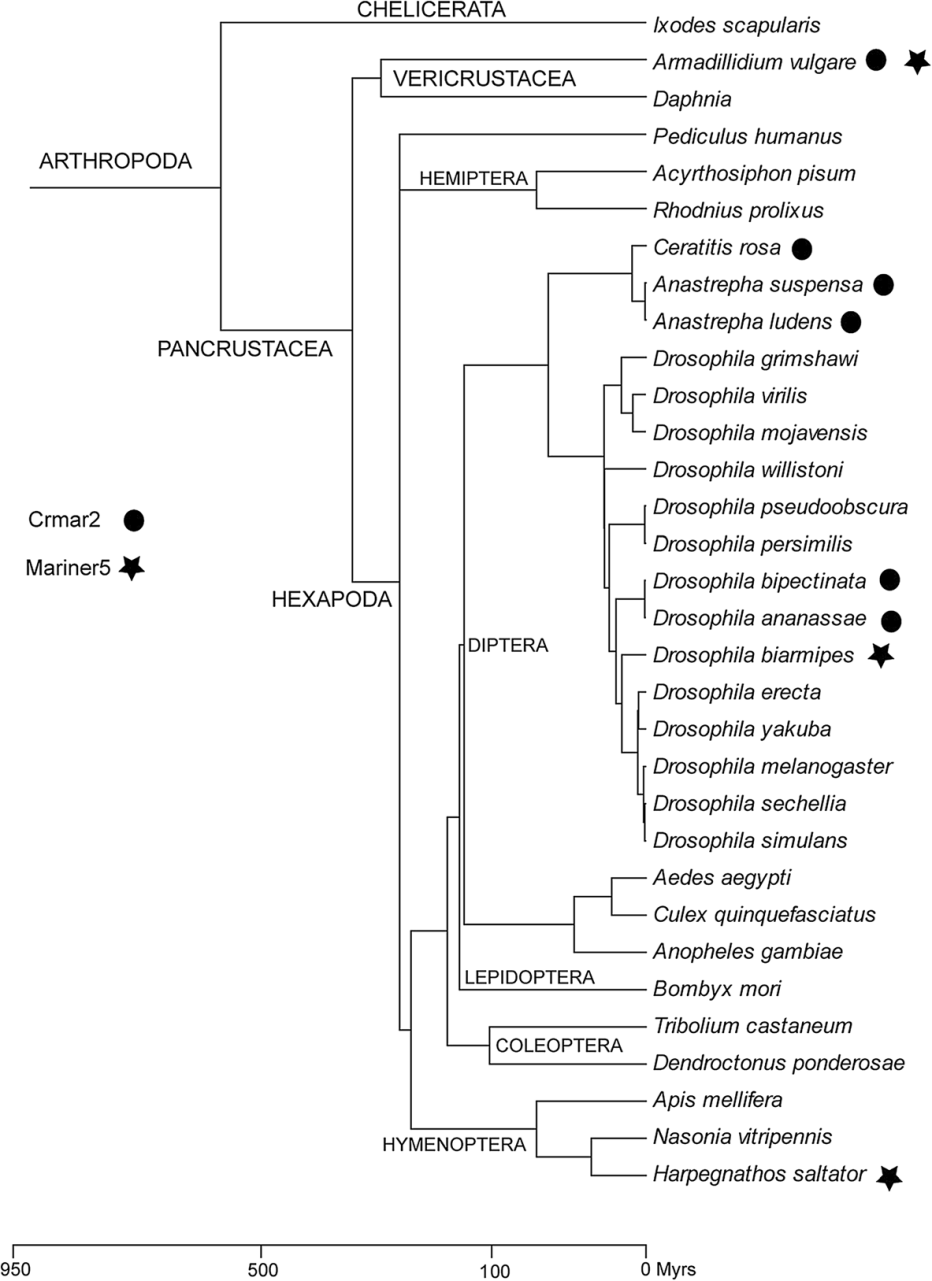
**Timetree of Arthropoda.** The tree includes all species in which *Crmar2* and *Mariner-5* were found as well as most closely related species for which whole genome sequences are available in Genbank. Phylogenetic relationships are taken from Tamura *et al.*[18], Regier *et al*. [19], Sharkey [20] and Regier *et al*. [7]. Divergence times are taken from Hedges *et al.*[21]. Divergence times between *D. bipectinata* and *D. ananassae*, between *D. biarmipes* and other *Drosophila* species, between *Dendroctonus ponderosae* and *Tribolium castaneum*, between *A. vulgare* and *Daphnia pulex,* and between *H. saltator* and *Nasonia vitripennis* are unknown and have therefore been set at arbitrary values for illustrative purposes.

Next we sought to assess the taxonomic distribution of *Crmar2_Avul* and *Mariner-5_Avul* in eukaryotes by performing BLASTn searches on all eukaryotic genomes that were available in Genbank as of May 2013. In addition to the species in which the two elements had previously been described (*C. rosa*, *Anastrepha ludens* and *Anastrepha suspensa* for *Crmar2*; *D. biarmipes* for *Mariner-5*_Dbi) we found TEs highly similar (>90% identity over >500 bp) to *Crmar2_Avul* in *Drosophila ananassae* and *Drosophila bipectinata*, and to *Mariner-5_Avul* in the ant *Harpegnathos saltator*. Interestingly, the taxonomic distribution of the two elements is patchy, not only at the level of the arthropod phylogeny, but also within the lower level taxa in which we found them (Figure 1), a pattern likely indicative of horizontal transfer [17]. For example, *Crmar2_Avul* is only present in two closely related *Drosophila* species out of the 13 that we searched, and *Mariner-5_Avul* was identified only in one of the three hymenopteran genomes available.

### Horizontal transfer of the two *Mariner* elements between hexapods and isopods and within hexapods

**Figure 2:**
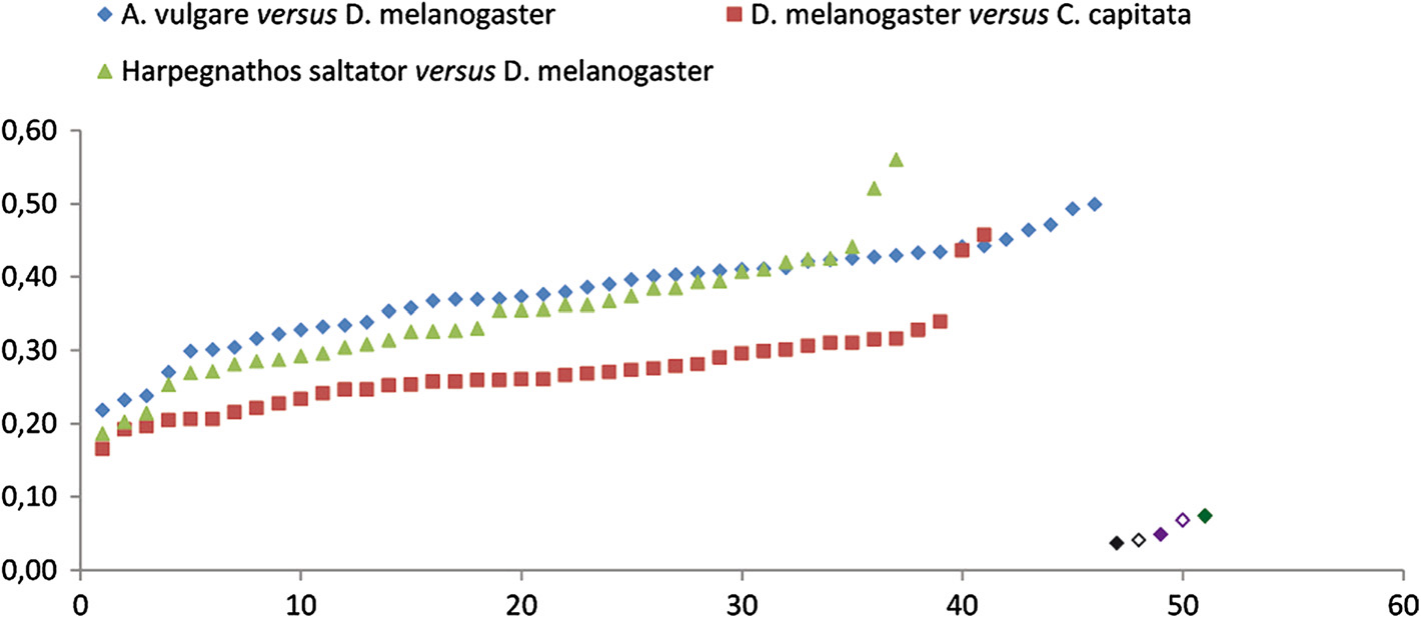
**Graph illustrating the pairwise corrected distances between arthropod orthologous genes and between*Crmar2*and*Mariner*-*5*consensus sequences.** Orthologous gene distances between *A. vulgare* and *D. melanogaster*, between *D. melanogaster* and *C. capitata*, and between *H. saltator* and *D. melanogaster* are illustrated with blue lozenges, red squares, and green triangles, respectively. Other symbols correspond to transposable element (TE) distances: *Mariner*-*5* between *H. saltator* and *D. biarmipes* (filled black lozenge), *Crmar2* between *C. rosa* and *D. bipectinata* (empty black lozenge), *Mariner*-*5* between *A. vulgare* and *D. biarmipes* (filled purple lozenge), *Crmar2* between *A. vulgare* and *C. rosa* (empty purple lozenge), and *Crmar2* between *A. vulgare* and *D. bipectinata* (filled green lozenge). A detailed list of genes and distances is provided in Additional file 8: Table S3. Before the distance values were plotted, they were sorted by ascending order.

To formally assess whether the distribution of the two elements in arthropods is the result of HT, we compared TE genetic distances calculated between hexapod species and *A. vulgare* to distances calculated for 46 orthologous genes available for both *A. vulgare* and *Drosophila melanogaster*[7]. As illustrated in Figure 2, distances between orthologous genes (average = 35%, min = 21%, and max = 49%) are much higher than distances between TEs (average = 6%, min = 4.9%, and max = 7.4%). Under vertical transmission of the TEs, TE distances between taxa are expected to be higher than distances between orthologous genes because TEs are known to evolve neutrally after insertion in a given genome [22], that is, faster than host genes that evolve under purifying selection due to functional constraints. The high similarity between *A. vulgare* and hexapoda TEs coupled with the deep divergence time between these two arthropod taxa (>400 million years) and to the patchy distribution of the two elements in arthropods allows us to confidently conclude that the presence of both *Crmar2* and *Mariner-5* in isopod crustaceans and hexapods results from HT. Interestingly, we found that gene distances between tephritid (*Ceratitis capitata*) and drosophilid (*D. melanogaster*) flies on one hand (average = 27%, min = 16%, and max = 45%) and between *Drosophila* and the ant *H. saltator* on the other hand (average = 34%, min = 18%, and max = 56%) are also much higher than TE distances (4% for both elements; Figure 2). This pattern suggests that in addition to transferring horizontally between hexapods and isopods, *Crmar2* also underwent HT between the two dipteran lineages and *Mariner-5* also transferred horizontally between dipterans and hymenopterans.

### Recent horizontal transfer of the two *Mariner* elements within isopods

**Figure 3:**
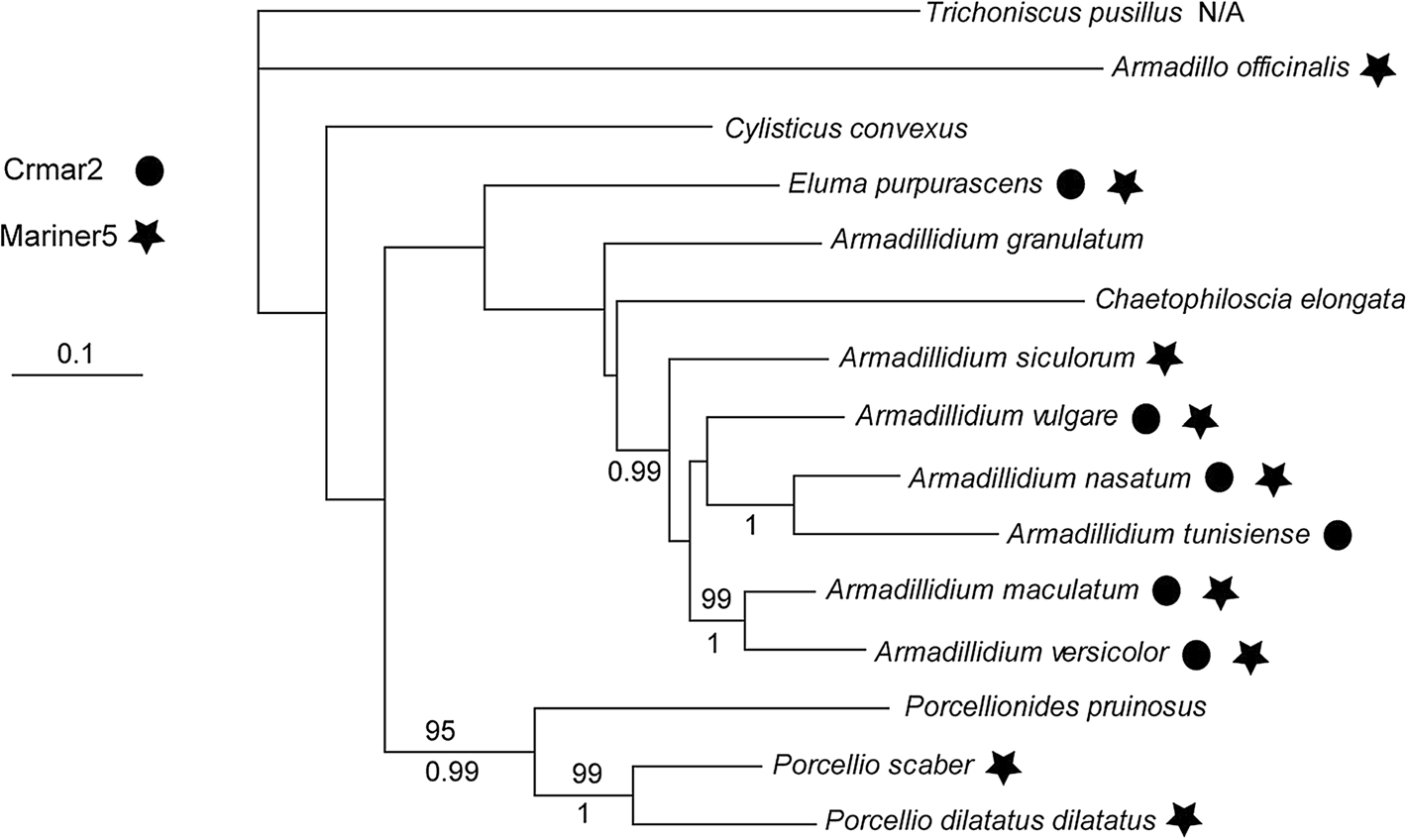
**Phylogenetic tree of the various terrestrial isopod crustacean species included in this study.** The topology of the tree corresponds to the consensus of the bootstrap analysis performed under the maximum likelihood criteria. Bootstrap values above 70% and Bayesian posterior probabilities above 0.9 are shown above and below branches respectively. Species in which *Crmar2* and/or *Mariner*-*5* were uncovered by PCR are marked with a filled circle and/or a star. *Trichoniscus pusillus* was used as an outgroup to root the present phylogeny based on the topology obtained in Michel-Salzat and Bouchon [23] and was not screened for the presence of the two TEs (N/A: not applicable). The other species were PCR screened but none of the two elements were amplified.

To shed light on the evolutionary dynamics of *Crmar2_Avul* and *Mariner-5_Avul* in terrestrial isopod crustaceans we carried out two sets of PCR screenings in 14 species. The first screening involved primer pairs designed in the internal region of the elements in order to check for the presence of each TE in the various species (type 1 primers in Additional file 2: Figure S2). The second screening aimed at finding specific copies of the two elements that would be shared at orthologous loci between the various isopod species. For the latter screen we used primer pairs for which one primer was designed in the 5’ or 3’ end of *Crmar2_Avul* and *Mariner-5_Avul* and the other primer was designed in the flanking region of several copies of each element (type 2 primers in Additional file 2: Figure S2; n = 4 for *Crmar2_Avul* and 3 for *Mariner-5_Avul*). The first screening (internal primers) uncovered *Crmar2_Avul* and *Mariner-5_Avul,* respectively, in six and nine of the 14 species (Figure 3). The second screening (search for orthologous copies) did not reveal any shared copies between *A. vulgare* and any of the other 13 isopod species. This absence of amplification could be due to a lack of conservation of the regions flanking the various copies in the different species. But perhaps more interestingly, we also noticed that two of the four *Crmar2* copies failed to amplify in one of the two *A. vulgare* individuals in which we searched them, suggesting that they are polymorphic in terms of presence/absence in *A. vulgare* populations. Overall, these data indicate that both elements likely underwent recent HT in isopods and may still be active.

**Figure 4:**
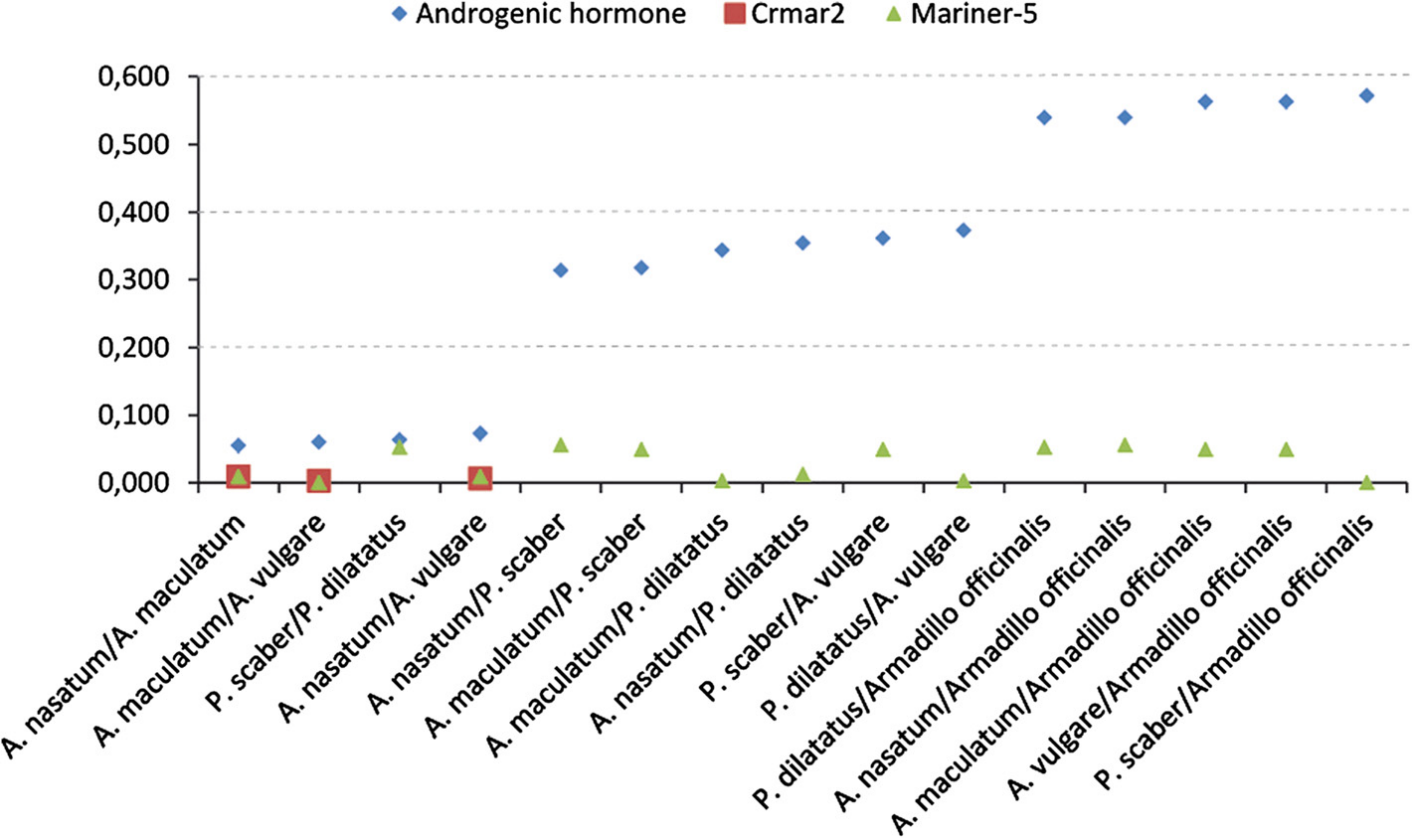
**Graph illustrating the pairwise corrected distances between the androgenic gland hormone gene and between** ***Crmar2*** **and** ***Mariner*-*5*** **consensus sequences within terrestrial isopods.**

To further test the possibility that both elements invaded isopod genomes recently via HT and are still actively transposing, we cloned and sequenced two to five different copies of *Crmar2_Avul* and *Mariner-5_Avul* in all isopod species in which we found them (except for *Eluma purpurascens* in which all ten clones that we sequenced contained an identical copy of *Crmar2*). Pairwise genetic distances between these copies within each genome are all very low (average = 1.2%, min = 0.9%, and max = 2% for *Crmar2_Avul* and average = 4.5%, min = 2%, and max = 6% for *Mariner-5_Avul*). In addition, the between-species distances for both TEs are also much lower than distances calculated for the androgenic gland hormone gene (average = 34%; Figure 4). Following the same reasoning as for the comparisons between TE and orthologous gene distances discussed above, we believe these results strongly suggest that both *Crmar2_Avul* and *Mariner-5_Avul* underwent one or more recent HTs within isopods. Interestingly, using RT-PCR, we verified that *Crmar2_Avul* and *Mariner-5_Avul* are transcribed both in somatic and germ cells in *A. vulgare* [see Additional file 3: Figure S3]. Furthermore, seven of the eight *Crmar2_Avul* transcripts that we uncovered in the *A. vulgare* transcriptome contain a full-length and intact (devoid of non-sense mutations) ORF (sequences provided in Additional file 4: Dataset 1), suggesting that at least one source of functional transposase is transcribed for this element in *A. vulgare*.

To provide an estimate of the absolute age of the activity burst of *Mariner*-*5* and *Crmar2*, we divided the average copy/consensus distance calculated for each element in *D. biarmipes* (11%) and *D. bipectinata* (6.9%) by the experimentally derived neutral substitution rate of *D. melanogaster* (0.0346 substitutions per base per million years (myr); [24-26]). This yielded a burst age of 3.2 myrs for *Mariner*-*5* in *D. biarmipes* and 2 myrs for *Crmar2* in *D. bipectinata*. The age of *Mariner-5_Avul* and *Crmar2_Avul* cannot be precisely estimated because nuclear substitution rates are not available for isopods. Together with the absence of shared orthologous copies of both elements between the various isopods species and the seemingly polymorphic state of *Crmar2* in *A. vulgare*, the fact that an intact *Crmar2_Avul* transposase is transcribed in this species is consistent with a recent invasion of isopod genomes by *Mariner-5_Avul* and *Crmar2_Avul* and suggest both elements are active sources of genomic variation in this major crustacean group. In addition, given that isopod *Crmar2* and *Mariner*-*5* are highly similar to *Drosophila Crmar2* and *Mariner*-*5* (95% and 93% identity, respectively; Figure 2), we believe these HTTs most likely took place within the past few million years at most.

### Number of horizontal transfer of transposon events

**Figure 5:**
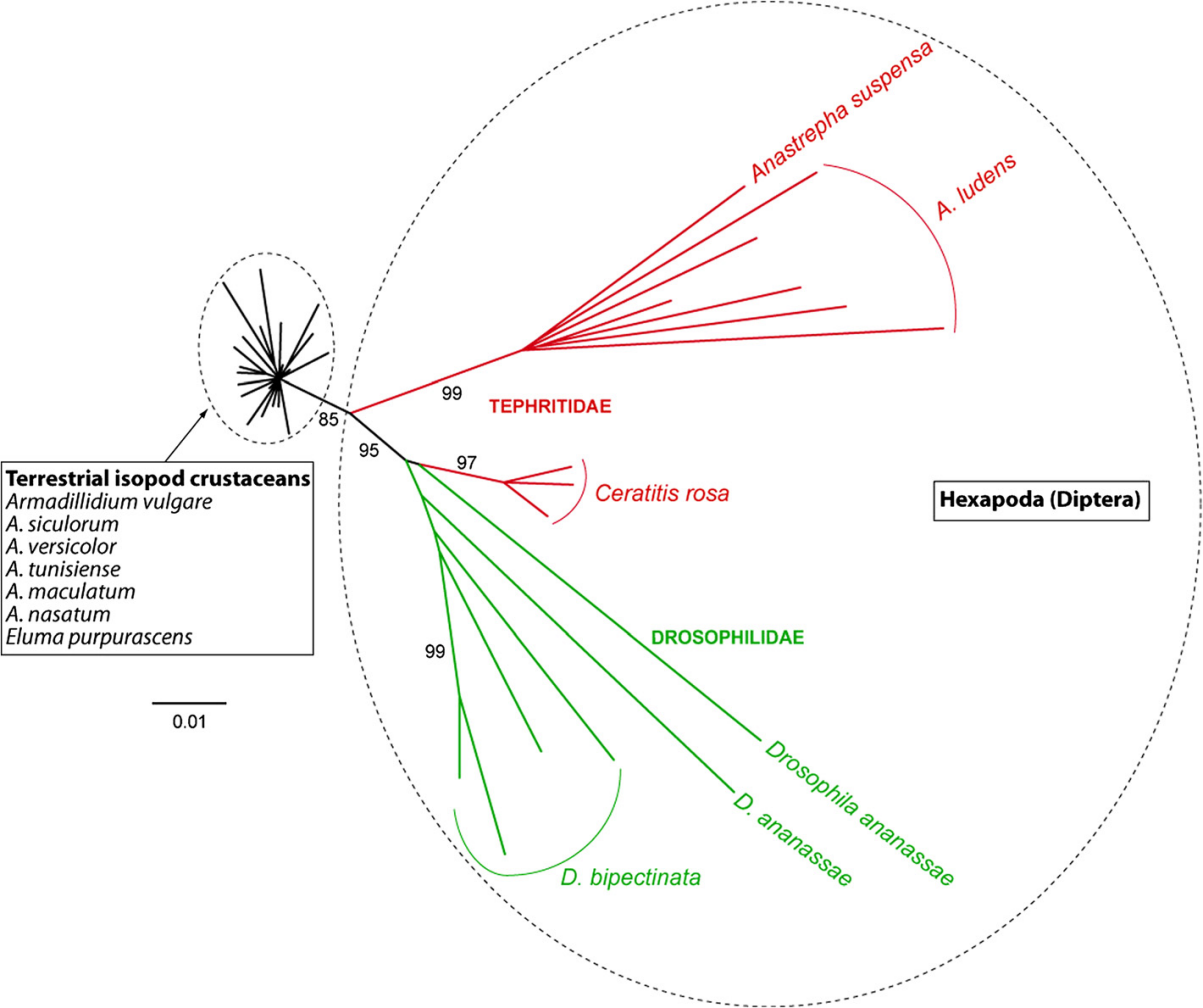
**Phylogenetic tree of*Crmar2*copies.** Maximum likelihood bootstrap values above 70% are shown on branches. Given the absence of phylogenetic support for the branching of *Crmar2* copies within isopods, the name of the species is shown only once in the black rectangle to facilitate the reading of the figure.

**Figure 6:**
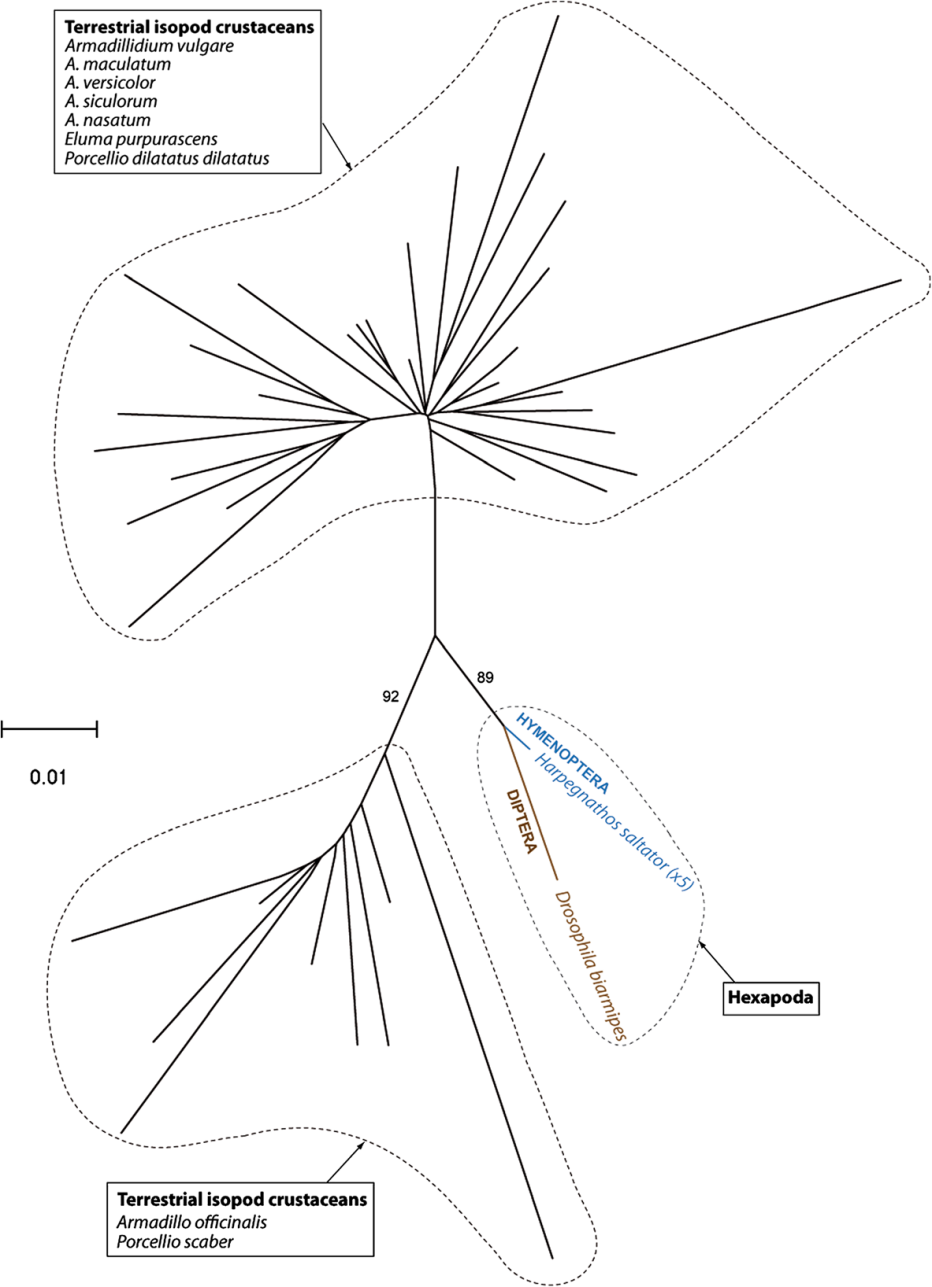
**Phylogenetic tree of*Mariner*-*5*copies.** Maximum likelihood bootstrap values above 70% are shown on branches. Given the absence of phylogenetic support for the branching of *Mariner*-*5* copies within the two clusters of isopod sequences, the name of the species is shown only once in the two black rectangles to facilitate the reading of the figure.

To assess the number of HTTs that occurred between hexapods and crustacean isopods and within each of the two taxa, we reconstructed a phylogeny of both elements based on an alignment including all copies of *Crmar2* and *Mariner-5* that we sequenced from the various isopod species and those that we found in the other sequenced arthropod genomes. In the resulting *Crmar2* tree both hexapod and isopod *Crmar2* elements are monophyletic (Figure 5). Therefore, a single HTT event between isopods and hexapods needs to be inferred to explain the taxonomic distribution of this element in arthropods. Within hexapods, *Crmar2* TEs from the tephritid *C. rosa* are more closely related to *Drosophila* elements than they are to the other tephritid elements found in *A. ludens* and *A. suspensa* by Gomulski *et al.*[11]. This topology upholds our TE versus orthologous gene distance analysis (see above), indicating that *Crmar2* also underwent HT within dipterans. In the *Mariner*-*5* tree (Figure 6), isopod elements fall within two relatively distantly related clusters. However, given that the tree is unrooted, we cannot conclude on whether *Mariner*-*5* was transferred once or more than once between hexapods and isopods. Interestingly, the topology of isopod *Mariner*-*5* copies (Figure 6) is clearly incongruent with that of the isopod tree (Figure 3). For example, *Porcellio dilatatus dilatatus* and *Porcellio scaber* form a strongly supported clade in the species phylogeny, but *Crmar2* copies from the former fall within the cluster that groups all *Armadillidium* and *Eluma purpurascens* copies and those of *P. scaber* group with *Crmar2* copies from *Armadillo officinalis*. This phylogenetic incongruence between host and TE phylogenies, together with the general lack of phylogenetic resolution within isopod *Crmar2* and *Mariner-5* clusters (Figures 5 and 6), and the fact that copies from each isopod species do not form monophyletic groups, further supports the HT of both elements between the various isopod species.

### Potential vectors of horizontal transfer

Though the question of the mechanisms and vectors underlying HTT between multicellular eukaryotes remains largely open, growing evidence suggests that host-parasite relationships likely facilitate such transfers [27-31]. In particular, viruses are often cited as ideal HTT vectors due to their capacity to inject DNA/RNA into host cells [32-35]. Though the viral fauna infecting the various species involved in *Crmar2* and *Mariner-5* HTT is poorly known, it is noteworthy that members of the Iridoviridae have been found in several species of dipterans, hymenopterans and terrestrial isopods [36,37]. In addition, a recent study identified two TEs inserted in the genome of an iridovirus infecting dipteran [38], emphasizing the potential of this type of viruses to shuttle transposons between their hosts. Another possible route for HTT to occur in arthropods is via transfers of endosymbiotic bacteria. Several species of isopods as well as *D. ananassae*, *D. bipectinata* and *A. suspensa* are known to bear intracellular, maternally transmitted alphaproteobacteria called *Wolbachia*[39-43]. The fact that isopod *Wolbachia* strains are known to have undergone several HT [44] and that several genes of eukaryotic origin have been found integrated in *Wolbachia* genomes [45-47] suggest that endosymbionts could also facilitate HT of DNA between hosts.

## Conclusions

In this study, we have characterized the evolutionary dynamics of two Tc1/Mariner elements in isopod crustaceans and shown that their current taxonomic distribution in arthropods results from at least one HT between hexapods and isopods as well as one or more HTs within isopods. Furthermore, we have demonstrated that *Crmar2* transferred horizontally between drosophilid and tephritid flies and that *Mariner-5* underwent HT between Diptera and Hymenoptera. Conservatively, and assuming that *Crmar2* and *Mariner-5* transferred horizontally only once within the isopods, we have uncovered a total of six new HTT events in this study. Together with 70 previously known cases (for example, [48-50]; reviewed in [4]) our results bring to 76 the number of HT events described in metazoans for the Tc1/Mariner superfamily, further emphasizing the indifference of these elements to host factors to transpose [51]. Of note, HT of two other *Mariner* elements have previously been characterized in marine crustaceans [52] (one between two decapods and one between a decapod and an amphipod), but our study is the first to report HTT involving terrestrial crustaceans. Finally, we have shown that these newly described HTT events most likely took place within the last 3 myrs, and we propose iridoviruses and/or *Wolbachia* endosymbionts as the potential vectors of transfer, a hypothesis that will be interesting to test in future studies.

## Methods

### Mining of available eukaryote genomes

In order to identify transposable elements similar to *Crmar2* and *Mariner-5_Dbi* that could have been horizontally transferred between isopods and other taxa we used the nucleotide sequence of the two elements to carry out BLASTn searches against the nr (non-redundant nucleotide), EST (expressed sequence tag) and WGS (whole genome sequence) databases available on the NCBI website. We considered only those elements that showed more than 90% nucleotide identity over more than 500 bp of our query sequences.

### DNA extraction, PCR, cloning, and sequencing

Genomic DNA was extracted from 14 species of terrestrial isopods (Figure 3) using the Qiagen™ DNeasy blood and tissue extraction kit (Hilden, Germany). PCRs were carried out using four types of primer pairs: 1) one pair designed on the internal region of *Mariner-5_Avul* and *Crmar2_Avul*, 2) three and four pairs designed to screen specific copies of the two elements at orthologous position in the 14 isopod species, 3) one pair designed to amplify the mitochondrial cytochrome oxidase I (*Co1*) for the six species for which this gene is not available in Genbank, and 4) one pair designed to amplify the mitochondrial 16S gene for all species included in this study except for *T. pusillus* (taken from Genbank). The list of PCR primers used in this study, together with their respective melting temperatures, is given in Additional file 5: Table S1. PCR reactions were conducted using the following temperature cycling: initial denaturation at 94°C for 5 min, followed by 30 cycles of denaturation at 94°C for 30 s, annealing at 52 to 58°C (depending on the primer pair) for 30 s, and elongation at 72°C for 45 sec, ending with a 10 min elongation step at 72°C. PCR products obtained with the *Co1* primers were purified and directly sequenced using ABI BigDye sequencing mix (1.4 μl template PCR product, 0.4 μl BigDye, 2 μl manufacturer supplied buffer, 0.3 μl primer, and 6 μl H2O). Sequencing reactions were ethanol precipitated and run on an ABI 3730 sequencer. PCR products obtained with the primers internal to *Crmar2_Avul* and *Mariner-5_Avul* were cloned into pGEM-T easy vector (Promega, USA, Madison, WI) and several clones were Sanger-sequenced as described above until we obtained five different copies of each element in the various species in which we found them.

### Sequencing of androgenic gland hormone (*Agh*) cDNA

Total RNA was isolated from androgenic glands of fifteen males (6 glands per individual) using the RNeasy Mini kit (Qiagen, Hilden, Germany). The cDNA was synthesized using the M-MLV-RT kit (Promega). PCR amplification was performed using several degenerated primer pairs designed on the consensus sequences of *Agh* cDNAs of *A. vulgare*, *P. scaber* and *P.dilatatus* [see Additional file 6: Table S2] [53]. PCR and direct sequencing were performed as described above.

### RT-PCR

The expression of *Crmar2_Avul* and *Mariner-5_Avul* was assessed in both dissected ovaries and somatic tissues (head + nervous chain) of *A. vulgare* females using the SuperScript™ III First-Strand Synthesis System for RT-PCR» (Invitrogen, Eugene, OR, USA).

### Transposon distances versus gene distances

All *Crmar2* and *Mariner-5* consensus sequences reconstructed in this study are provided in Additional file 7: Dataset 2. In order to test whether *Crmar2* and *Mariner-5* TEs were inherited vertically or horizontally we compared the distances calculated between TE consensus sequences and between several orthologous genes for several pairs of taxa. To calculate gene distances between Isopoda and Hexapoda, we used the 57 *A. vulgare* genes sequenced by Regier *et al*. [7] as queries to perform BLASTn searches against the *D. melanogaster* genome. We chose the *D. melanogaster* genome rather than the genome of *Drosophila* species involved in the HTT characterized in this study because it is the most completely sequenced and best assembled *Drosophila* genome available. We found 46 *A. vulgare* orthologs in *D. melanogaster*, which we aligned at the nucleotide level between the two species. For the *Ceratitis*/*Drosophila* gene distances we used the 46 genes resulting from the above search as queries to perform BLASTn searches against the whole genome sequence of *C. capitata*. This search yielded 41 genes that we aligned at the nucleotide level with those of *D. melanogaster*. The same approach was used to find genes orthologous between *D. melanogaster* and *H. saltator* which resulted in the alignment of 37 genes. Genetic distances between *Crmar2* and *Mariner-5* consensus sequences as well as between each pair of orthologous genes were calculated using the Jukes Cantor model in MEGA 5 [54]. The name of all genes and the distances between them are provided in Additional file 8: Table S3. Jukes Cantor distances were also calculated between the various copies of both TEs found within each genome in which we found them.

### Phylogenetic analyses

The phylogeny of the 14 terrestrial isopod species understudy was reconstructed using *16S*, *Co1*, and *Agh* sequences. All sequences produced in this study were deposited in Genbank under accession numbers KF957774-KF957833. Each gene was aligned manually using BioEdit 7.0.5.3 [55], and ambiguous regions were removed. All alignments are provided in Additional file 9: Dataset 3. A bootstrapped neighbor joining phylogeny was first reconstructed using MEGA 5 for each alignment with the maximum likelihood distance option. Given that no incongruence supported by bootstrap values >50% was observed between the three resulting trees (not shown), we then concatenated the three alignments and reconstructed a bootstrapped maximum likelihood phylogeny of the three combined markers using PhyML 3 [56]. A bayesian analysis of this alignment was also performed using MrBayes [57] in order to obtain posterior probabilities for each node of the tree. The model of nucleotide evolution best fitting the combined alignment (GTR + I + G) and used for the phylogenetic analyses was chosen based on the Akaike information criterion (AIC) in jModeltest 2 [58].

The phylogeny of *Crmar2* and *Mariner-5* was reconstructed based on alignments of all different copies of each element from each species in which we found them. Ambiguous regions and regions absent in more than 25% of the sequences were removed. Alignments are provided in Additional file 9: Dataset 3. The model of nucleotide evolution best fitting each alignment (TPM1uf + G for both elements) was chosen based on the Akaike information criterion (AIC) in jModeltest 2 and each alignment was analyzed using PhyML 3. In order to assess the phylogenetic position of *Mariner-5_Dbi* in the mariner tree, we have aligned the amino acid sequence of a transposase representative of most described mariner subfamily and performed a neighbor joining analysis using MEGA 5 (JTT model, 1000 bootstrap replicates). The accession numbers of the sequences we have used are provided in Additional file 1: Figure S1.

## Abbreviations

HTT: horizontal transfer of transposons; LTR: long terminal repeat; myr: million year; RT-PCR: reverse-transcription polymerase chain reaction; TE: transposable element.

## Competing interests

The authors declare that they have no competing interests.

## Authors’ contributions

MD and CG designed the study, carried out the experiments and analyses and drafted the manuscript. SL generated the draft assembly of the *Armadillidium vulgare* genome and contributed to the writing of the manuscript. NC sequenced the androgenic gland hormone gene in the various isopod species and contributed to the writing of the manuscript. DB provided the draft transcriptome assembly of *A. vulgare* and contributed to the writing of the manuscript. All authors read and approved the final manuscript.

## Supplementary Material

Additional file 1: Figure S1 Phylogenetic relationships between various mariner transposases showing that *Mariner-5_Dbi* belongs to the *irritans* subfamily. Click here for file

Additional file 2: Figure S2 Illustration of the position of the two types of primer sets we used to screen for *Mariner-5_Avul* and *Crmar2_Avul* elements in the various isopod species. The sequence of the primers is provided in Additional file 5: Table S1. TIR: Terminal inverted repeat. Click here for file

Additional file 3: Figure S3 Pictures of agarose gels showing the results of the reverse transcription PCR experiments on *Crmar2 ***(A)** and *Mariner*-*5***(B)** in *Armadillidium vulgare* ovaries and somatic tissues (head + nervous chain). A band of the expected size was obtained for all reactions showing that both elements are transcribed in *A. vulgare* soma and germ line. RT, reverse transcriptase; L, size ladder. Click here for file

Additional file 4: Dataset 1 Sequences of the *Crmar2* transcripts encoding a full length, intact, and thus potentially functional transposase aligned together with the *Crmar2_Avul* consensus sequence (fasta format). Click here for file

Additional file 5: Table S1 List of primers used to amplify and sequence *Crmar2* and *Mariner*-*5* elements. Click here for file

Additional file 6: Table S2 List of primers used to amplify and sequence the androgenic gland hormone. Click here for file

Additional file 7: Dataset 2 Consensus sequences of *Crmar2* and *Mariner*-*5* elements reconstructed in this study (fasta format). Click here for file

Additional file 8: Table S3 List of genes sequenced by Regier *et al*. [7] used to calculate genetic distances between the various species included in this study. Click here for file

Additional file 9: Dataset 3 Sequence alignments used to reconstruct the phylogeny of crustacean isopod species and *Crmar2* and *Mariner*-*5* copies (fasta format). Click here for file
