# Supplementary figures and images for: Horizontal transfer of transposons between and within crustaceans and insects
Source: Mob DNA. 2014 Jan 29;5:4. doi: 10.1186/1759-8753-5-4 (PMC3922705; doi:10.1186/1759-8753-5-4)

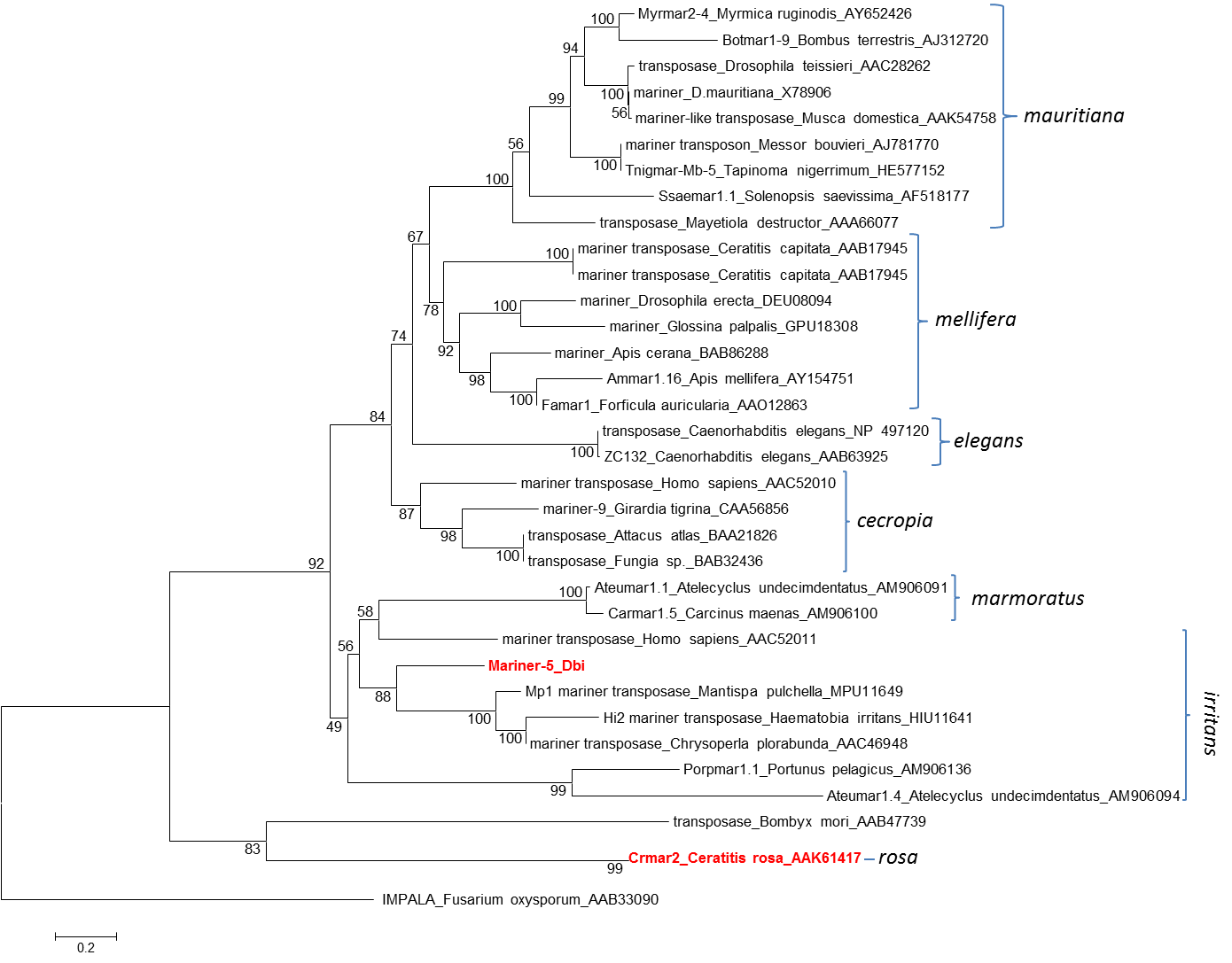

Supplement: Additional file 1: Figure S1 — Phylogenetic relationships between various mariner transposases showing that Mariner-5_Dbi belongs to the irritans subfamily. [file 1759-8753-5-4-S1.tiff]

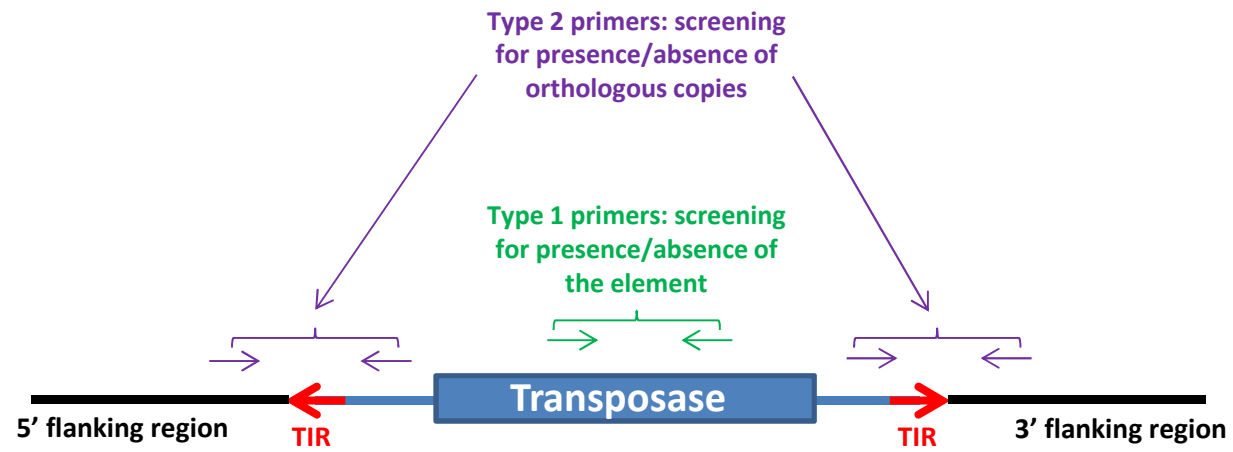

Supplement: Additional file 2: Figure S2 — Illustration of the position of the two types of primer sets we used to screen for Mariner-5_Avul and Crmar2_Avul elements in the various isopod species. The sequence of the primers is provided in Additional file 5: Table S1. TIR: Terminal inverted repeat. [file 1759-8753-5-4-S2.pdf]

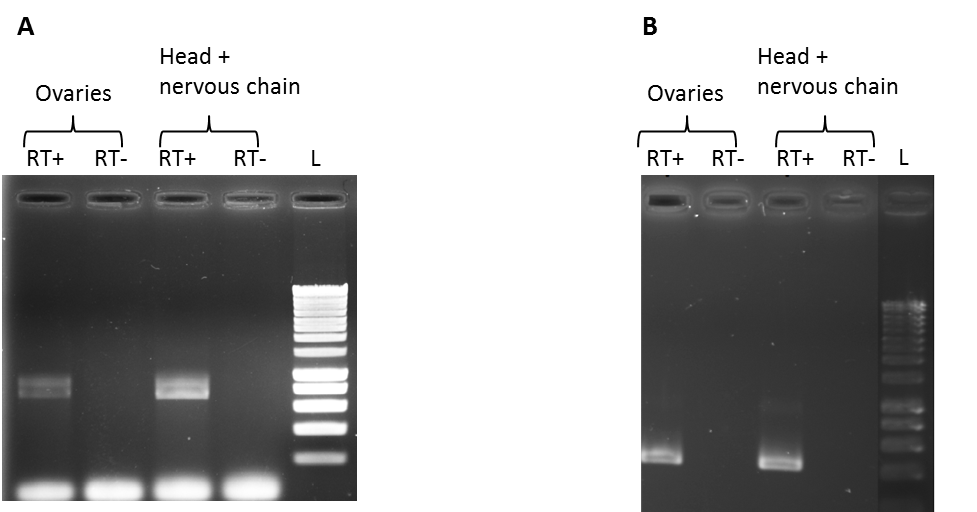

Supplement: Additional file 3: Figure S3 — Pictures of agarose gels showing the results of the reverse transcription PCR experiments on Crmar2 (A) and Mariner-5(B) in Armadillidium vulgare ovaries and somatic tissues (head + nervous chain). A band of the expected size was obtained for all reactions showing that both elements are transcribed in A. vulgare soma and germ line. RT, reverse transcriptase; L, size ladder. [file 1759-8753-5-4-S3.tiff]
