# Supplementary material for: Horizontal transfer of transposons between and within crustaceans and insects
Source: Mob DNA. 2014 Jan 29;5:4. doi: 10.1186/1759-8753-5-4 (PMC3922705; doi:10.1186/1759-8753-5-4)
Supplement: Additional file 5: Table S1 — List of primers used to amplify and sequence Crmar2 and Mariner-5 elements. [file 1759-8753-5-4-S5.pdf]

Name of forward primer	Sequence of forward primer	Name of reverse primer	Sequence of reverse primer	Usage
F785	GTACGTGCCCTTAAGACCAG	R785	TCACGAAGTGCACGAAATAC	Screening for orthologous copies of Crmar2_Avul in various terrestrial isopod crustacean species
F593	TCCATACCTTCCAATTCGTGC	R593	CGTACTCTGCTTTTATCGAGTC	
F893	CGATTGTACGCACATTTGGACG	R893	CAGTCTATATCTCTTACCTGGTATTTAG	
F712	CATTGCCAATCCTTGCCAATC	R712	CAGTAAAGTGATAAGGTAAAGTAA	
F866	GAGTCGAATGAGCAGGTCATC	R866	ATTAAAGCAGGCGGTACGTG	Screening for orthologous copies of Mariner-5_Avul in various terrestrial isopod crustacean species
F174	GTACTTATCGGACTGCCCTC	R174	CAGCACATACGATGGACTATG	
F002	GTAGCCATCTACGAGAATGTAC	R002	CTGTTGGTCGCTCATCAAC	
Crmar2_Avul_F	TTAATCGTCCAAATGTGCGTAC	Crmar2_Avul_R	AGGGCCGATGTTGATTTTGA	Screening for presence/absence of Crmar2_Avul (internal primers)
Crmar2_Avul_F2	CGTCGTTGATGAACATTATGC	Crmar2_Avul_R2	GGTTCTATGCCGTTAATGTGC	
M5F2	CTATGCCGAATTATTGGGC	M5R2	CACTTGCCCCAGCGATG	Screening for presence/absence of Mariner-5_Avul (internal primers)
