# Supplementary material for: Horizontal transfer of transposons between and within crustaceans and insects
Source: Mob DNA. 2014 Jan 29;5:4. doi: 10.1186/1759-8753-5-4 (PMC3922705; doi:10.1186/1759-8753-5-4)
Supplement: Additional file 6: Table S2 — List of primers used to amplify and sequence the androgenic gland hormone. [file 1759-8753-5-4-S6.doc]

**Supplementary Table 2**

| **Targets** | **Names** | **Sequences** | **Tm** | **F/R** |
| --- | --- | --- | --- | --- |
| Androgenic Gland | AGH 331 | GTCCATTACGAAAAGAACTAAAG | 62 | F |
| Hormone | AGH 100* | CTAAAGTAGTGTCRAAATTWTTATAAAC | 68 | F |
|  | AGH 52 | GTAGTGTCAAAATTTTTATAAAC | 56 | F |
|  | AGH 35 | ACTAACATGAAGGGTCTCGTCAT | 66 | F |
|  | AGH -Po F1* | AYAAAWATGAAAGGTCTCCTCTTC | 66 | F |
|  | AGH 108* | ATGAARGGTCTYSTCWTCWTA | 58 | F |
|  | AGH 101* | TACCAGGTAVDAGGTATGARATC | 68 | F |
|  | AGH 104* | GATYTCATACCTHBTACCTGGTA | 68 | R |
|  | AGH 109* | TGARRTCBGATGTVWWMTGYGSRGA | 62 | F |
|  | AGH 102* | TGTATATGCAACGAATTRGG | 56 | F |
|  | AGH 105* | CCYAATTCGTTGCATATACA | 56 | R |
|  | AGH 110* | CWTGYCCWTGGYCHMMSAG | 56 | F |
|  | AGH 111* | CTSKKDGRCCAWGGRCAWG | 56 | R |
|  | AGH 28 | CAGAGGGAATACTCAGTGCCCGTGGATG | 88 | R |
|  | AGH 103 | AAGARTGTTGCAAYATTMGRAC | 60 | F |
|  | AGH 106* | GTYCKAATRTTGCAACAYTCTT | 60 | R |
|  | AGH 112* | TTCKRCARTAVARKKMHACWRTWGT | 58 | R |
|  | AGH 113* | TYYMWNSKYBWRWATBTTCKRCARTA | 58 | R |
|  | AGH 107* | CGCGTTCAGTGAAATTCCAA | 58 | R |
|  | AGH 32 | AAAAGCTTAATTAAATTGCCCCAAAAATGC | 78 | R |
|  | AGH -Po R2* | TTTTGTTCTAACAAARAAGATTATRAC | 64 | R |
|  | AGH 56 | CTCCAAAGAATTTATTAATAAC | 54 | R |
|  | AGH -Po R3* | TTTGAKGTTTGAACWWTGACAATTTTC | 68 | R |
|  | AGH -Pg_S | CGATTATGAATAGAAAATTCGC | 58 | R |
|  | AGH -Pg_L | GCGATTATGAATAGAAAATTTAT | 56 | R |

Supp_data-AH1: List of primers used for sequencing or for Androgenic Gland Hormone cDNA amplifications. * degenerated primers according to the International Union of Biochemistry (IUB) code. The melting temperature (TM) is in Celsius degrees. F: Forward primers and R: Reverse primers.
