# Supplementary material for: Horizontal transfer of transposons between and within crustaceans and insects
Source: Mob DNA. 2014 Jan 29;5:4. doi: 10.1186/1759-8753-5-4 (PMC3922705; doi:10.1186/1759-8753-5-4)
Supplement: Additional file 8: Table S3 — List of genes sequenced by Regier et al. [7] used to calculate genetic distances between the various species included in this study. [file 1759-8753-5-4-S8.pdf]

D. melanogaster - A. vulgare	
**Genes**	**Distances**
Casein kinase	0,218
ATP synthase	0,232
Alpha-spectrin 2	0,238
Protein kinase	0,27
mRNA splicing spliceosome associated protein	0,299
Prohormone convertase	0,301
Acétyl-glucosaminyl transferase Super sexcombs	0,304
ARN pol	0,316
Alpha spectrin	0,322
Neurofibromine	0,328
Proteasome subunit	0,332
Histone deacetylase	0,334
Arginin methyl-transferase	0,338
RNA pol II	0,353
Glucose phosphate dehydrogenase	0,358
F-box protein	0,367
Alpha adaptin	0,369
Myosin	0,369
Clathrin heavy chain	0,37
ATP dependent Helicase activity RNA helicase	0,373
ATPase	0,376
pre-mRNA processing factor 8	0,379
Triose phosphate isomerase	0,386
Elongation factor 1 alpha	0,39
RNA pol II largest subunit	0,396
Elongation factor 2	0,401
Carmine (clathrin adaptor)	0,403
ATP dependent helicase activity RNA helicase	0,405
protéine transmembranaire	0,408
Protein kinase 1	0,41
Shibire (dynamin)	0,411
Clathrin heavy chain 2	0,412
Protein phosphatase	0,421
Burgundy Glycine amidotransferase	0,423
RNA pol II largest subunit (autre)	0,425
pre-mRNA splicing factor	0,427
Replication licensing factor	0,429
Oscillin glucose phosphate isomerase	0,433
Acetyl coenzyme A carboxylase	0,434
Alpha-spectrin	0,441
Myosin	0,442
Leucine tRNA synthetase	0,451
Alanine tRNA synthetase	0,464
Glycogene synthase	0,471
Pugilist E tetrahydropholate synthetase	0,493
Integrin-linked kinase	0,499
	**Average**
	0,379
	**Min**
	0,218
	**Max**
	0,499

D. melanogaster - C. capitata	
**Genes**	**Distances**
Neuroendocrine convertase II	0,165
Ca/Calmodulin-dependent protein kinase 2	0,192
ATPase B vacuole	0,196
glucose-6-phosphate 1-dehydrogenase	0,204
Casein kinase 2 alpha subunit	0,206
Translation elongation factor II	0,206
protein phosphatase PP2A 55kDa regulation subunit	0,215
Oscillin (N-glucosamine-6-phosphate isomerase)	0,221
Shibire dynamin	0,227
Clathrin heavy chain F	0,233
DNA-directed RNA pol II subunit RPB1 (plus long)	0,241
Alpha adaptin A (Cc : P2 subunit alpha)	0,246
RNA pol II	0,246
Super sexcombs B (Cc : UDP N-acetylglucosamine)	0,252
pre-mRNA splicing processing factor 8	0,253
Clathrin heavy chain F 2	0,257
Myosin	0,257
ATPase vacuole H+	0,259
RNA pol II	0,259
pre-mRNA processing factor 8	0,26
DNA-directed RNA pol II subunit RPB1	0,26
Alpha spectrin	0,266
Fbox 11	0,268
Rpd3 histone deacetylase	0,27
mRNA splicing (Cc : splicing factor 3B subunit 1)	0,273
ATP dependent helicase activity	0,275
Burgundy (Cc : GMP synthase)	0,278
ATP dependent helicase activity	0,281
Myosin	0,29
Leu tRNA	0,296
Integrin linked protein kinase	0,299
Carmine (Cc : AP3 complex subunit mu 2)	0,301
elongation factor 1-alpha 48D	0,306
Alanine tRNA synthetase	0,31
Alpha spectrin D	0,31
Replication licensing factor	0,315
Arginine methyl-transferase 1	0,316
Glycogen synthase activity	0,328
cAMP-depdt protein kinase type I regulation subunit	0,339
Pugilist E (Cc : c-1-tetrahydrofolate synthase cytoplasmic	0,436
transmembrane 9 superfamily member2	0,457
	**Average**
	0,270
	**Min**
	0,165
	**Max**
	0,457

D. melanogaster - H. saltator	
**Genes**	**Distances**
ATPase B vacuole	0,186
gluc-6-P 1-dehydrogenase	0,202
Elongation factor 1 alpha 48D	0,214
Myosin	0,253
pre-mRNA splicing processing factor 8	0,269
Casein kinase 2 alpha subunit	0,271
Glycogene synthase activity	0,281
F-box 11	0,285
Rpd3 histone deacetylase	0,287
ATP depdt helicase activity	0,292
DNA-directed RNA pol II subunit RPB1 (plus long)	0,296
RNA pol II	0,304
Clathrin heavy chain F 2	0,308
phosphatase protein kinase type 1	0,314
pre-mRNA processing factor	0,325
mRNA splicing	0,326
DNA-directed RNA pol II subunit RPB1	0,327
RNA pol II	0,33
Super sexcombs B	0,354
Alpha-spectrin D	0,355
Arginine methyl-transferase 1	0,356
Alpha adaptin A	0,362
Clathrin heavy chain F	0,362
ATPase vacuole H+	0,367
Leu tRNA	0,374
Oscillin	0,384
transmembrane 9 superfamily member2	0,385
protein phosphatase PP2A 55kDa regulation subunit	0,393
Neurofibromin 1	0,394
Neuroendocrine convertase II	0,407
insert cDNA	0,41
Myosin	0,42
Replication licensing factor	0,424
Pugilist E	0,425
Integrin linked-kinase	0,441
triose phosphate isomerase	0,521
Burgundy	0,56
	**Average**
	0,345
	**Min**
	0,186
	**Max**
	0,560
